# Obesity-Associated Vitamin D Deficiency Correlates with Adipose Tissue DNA Hypomethylation, Inflammation, and Vascular Dysfunction

**DOI:** 10.3390/ijms232214377

**Published:** 2022-11-19

**Authors:** Imaduddin Mirza, Ariej Mohamed, Hania Deen, Swetha Balaji, Duaa Elsabbahi, Amier Munasser, Dina Naquiallah, Uzma Abdulbaseer, Chandra Hassan, Mario Masrur, Francesco M. Bianco, Mohamed M. Ali, Abeer M. Mahmoud

**Affiliations:** 1 Division of Endocrinology, Diabetes, and Metabolism, Department of Medicine, College of Medicine, The University of Illinois at Chicago, Chicago, IL 60612, USA; 2 Department of Surgery, College of Medicine, The University of Illinois at Chicago, Chicago, IL 60612, USA; 3 Department of Physical Therapy, College of Applied Health Sciences, The University of Illinois at Chicago, Chicago, IL 60612, USA; 4 Department of Kinesiology and Nutrition, College of Applied Health Sciences, The University of Illinois at Chicago, Chicago, IL 60612, USA

**Keywords:** adipokines, adipose tissues, DNA methylation, inflammation, obesity, vascular dysfunction, vitamin D deficiency

## Abstract

Vitamin D (VD) deficiency is a hallmark of obesity and vascular dysfunction. We sought to test the hypothesis that VD deficiency may contribute to obesity-related vascular dysfunction by inducing adipokine hypomethylation and augmented expression. To this end, we collected blood and adipose tissues (ATs) from a cohort of 77 obese participants who were classified as having mild, moderate, or severe VD deficiency. The body composition, vascular reactivity, cardiometabolic profiles, and DNA methylation of 94 inflammation-related adipokines were measured. Our results show that higher degrees of VD deficiency were associated with lower DNA methylation and induced the expression of inflammatory adipokines such as B-cell lymphoma 6 (*BCL6*), C-X-C Motif Chemokine Ligand 8 (*CXCL8*), histone deacetylase 5 (*HDAC5*), interleukin 12A (*IL12A*), and nuclear factor κB (*NFκB*) in the ATs. They were also associated with higher BMI and total and visceral fat mass, impaired insulin sensitivity and lipid profiles, AT hypoxia, and higher concentrations of circulating inflammatory markers. Moderate and severe VD deficiency correlated with impaired vasoreactivity of the brachial artery and AT-isolated arterioles, reduced nitric oxide generation, and increased arterial stiffness. In a multivariate regression analysis, the VD deficiency level strongly predicted the adipokine methylation score, systemic inflammation, and microvascular dysfunction. In conclusion, our findings suggest that VD deficiency is a possible contributor to obesity-related adipokine hypomethylation, inflammation, and vascular dysfunction.

## 1. Introduction

Over a third of the world’s population is affected by obesity and VD deficiency, both of which are significant public health problems. Obese people’s serum VD levels were found to be lower than those of people with average body weight [1]. Several hypothesized causes for the widespread VD deficiency among the obese have been proposed. Vitamin D is produced in the skin in response to sunlight’s ultraviolet radiation [2]. Accordingly, low VD levels have been linked to inactivity, specifically a lack of outdoor activity, which may explain why obese people tend to have VD deficiency. Due to VD sequestration within the fat, its bioavailability is thought to be lesser in obese people than in lean individuals [3]. Finally, VD deficiency can arise from poor gut absorption or a diet too low in VD-rich foods [4,5].

While VD deficiency has been linked to obesity, its role in the increased risk of obesity-associated cardiovascular disease has not been well identified. Several cardiovascular diseases, including left ventricular hypertrophy, hypertension, atherosclerosis, and vascular dysfunction, have been associated with VD deficiency, but the mechanisms underlying these associations are still largely unknown [4,6,7,8,9,10,11,12]. The most compelling epidemiological evidence comes from studies showing a connection between VD deficiency and eclampsia, a condition marked by hypertension [13]. Intervention studies corroborated these findings, showing that VD supplementation decreased arterial stiffness and mean blood pressure (2000 IU/day for two weeks) [14] and increased renal blood flow in response to angiotensin II infusion (15,000 IU/day for four weeks) [15].

While oxidative stress, Ca^2+^ signaling, and impaired nitric oxide (NO) production have all been implicated as contributing to the impact of VD deficiency on vascular function, inflammation ranks at the top [16,17]. Inflammatory cytokines play a central role in the pathogenesis of vascular dysfunction and cardiovascular diseases, and VD is commonly regarded to have a role in modulating inflammation by regulating the production of inflammatory cytokines [18]. Vitamin D deficiency was also linked to the development of several chronic inflammatory diseases other than cardiovascular diseases, such as inflammatory bowel syndrome, nonalcoholic fatty liver disease (NAFLD), chronic obstructive pulmonary disease (COPD), and others [19]. On the other hand, the cytokine profiles of patients with congestive heart failure and other cardiovascular disorders were demonstrated to be improved by VD administration [20]. Additionally, our recent studies showed improvements in the flow-induced dilation of AT arterioles in response to VD treatment [21]. These and other studies suggest that inflammation and an increased production of inflammatory cytokines are at the root of the link between VD deficiency and vascular dysfunction.

Our recent research established a connection between vascular dysfunction and the hypomethylation of inflammatory adipokine genes [22,23]. It is important to note that the degree of methylation of a gene is inversely proportional to its level of expression, with lower methylation indicating greater expression. Therefore, we sought to investigate the association between VD deficiency and adipokine methylation in the adipose tissue (AT) of obese individuals to better understand the mechanisms by which VD deficiency predisposes to vascular dysfunction. Vitamin D performs as a transcription factor through its receptor, the vitamin D receptor (VDR) [24]. Recently, VD’s role in modulating gene expression through the regulation of DNA methylation has been established as yet another mechanism. Researchers have found that VD levels correlate with the methylation status of genes critical for VD metabolism and other developmental and metabolic pathways [25,26,27]. However, a dearth of studies directly examines these correlations with adipokine methylation, particularly in the context of obesity. Current research investigates DNA methylation of inflammatory cytokines produced by the AT (adipokines) in obese participants classified as having mild, moderate, or severe VD deficiency. In addition to the vasoreactivity of arterioles isolated from adipose tissue, several anthropometric and cardiometabolic variables were also evaluated. The central hypothesis is that VD deficiency plays a role in vascular dysfunction via mechanisms that may include the epigenetic upregulation of inflammatory adipokines.

## 2. Results

### 2.1. Anthropometric and Cardiometabolic Measures

**Figure 1:**
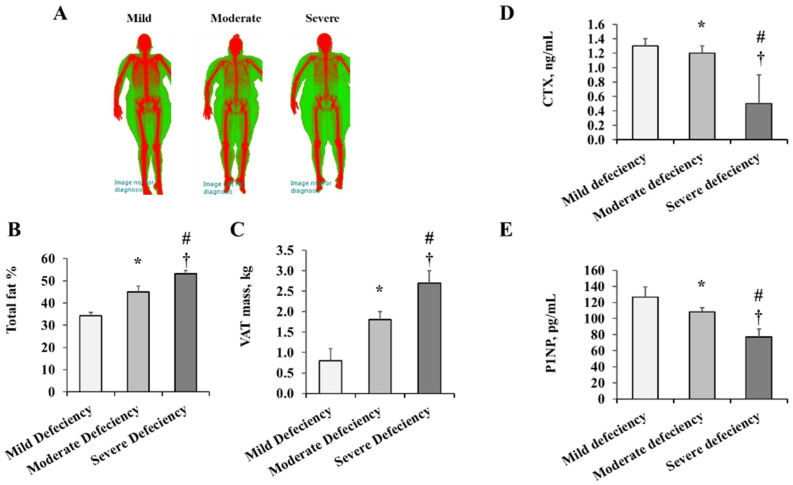
Fat percentage and bone turnover enzymes across VD deficiency categories. (**A**) Representative images of DEXA-measured body composition. Graphical representations of total fat % (**B**) and VAT mass (**C**) across VD deficiency groups. Serum levels of CTX (**D**) and P1NP (**E**) measured by ELISA assays. Results are represented as means ± standard error (SE). A significant *p*-value is denoted by * for the comparison between the mild and moderate deficiency groups, # for the comparison between the mild and severe deficiency groups, and † for the comparison between moderate and severe deficiency groups.

Table 1 summarizes VD measurements, body composition, and bone mass measurements. All the study participants were VD deficient and were classified into mild VD deficiency (12–19 ng/mL, *n* = 21), moderate VD deficiency (5–11 ng/mL, *n* = 30), and severe VD deficiency (<5 ng/mL, *n* = 26). Moderate and severe VD deficiency categories had significantly higher body weight and BMI than the mild VD deficiency category. As measured by DEXA, the percentages of total, android, and visceral fat increased with increasing VD deficiency, with severe deficiency exhibiting the highest fat percentages. Figure 1A illustrates the body fat versus lean composition measured by DEXA scanning. When quantified (Figure 1B), the total fat percentage was 27% and 44% higher in the moderate and severe VD deficiency groups, respectively, compared to the mild category. Similarly, VAT mass was 77% and 1.5-fold higher in individuals with moderate and severe VD deficiency, respectively, compared to those with mild deficiency (Figure 1C). Compared to males, females were found to have higher total fat percentage (mean: M = 45.9, F = 53.5, *p* = 0.0013) and VAT percentage (mean: M = 52.5, F = 45.4, *p* = 0.0013). Although African American (AA) participants had an 8% to 10% higher BMI and total and visceral fat percentages than white participants, this difference did not reach statistical significance. Based on this potential impact, sex and race were both adjusted for the in-between group analyses.

Bone mineral density (BMD) and content (BMC) were paradoxically higher in people with severe VD deficiency compared to those with mild and moderate deficiency (Table 1). Other DEXA-measured bone mass parameters included the T-score (bone density relative to that of a healthy 30-year-old of the same gender) and the Z-score (bone density comparable to that of an average person of the same age and gender). Those with severe VD deficiency had the highest T and Z scores. In addition, C-terminal telopeptide of type 1 collagen (CTX), a bone resorption enzyme, and procollagen type 1 N propeptide (P1NP), a bone formation enzyme, were determined. These enzymes were markedly lower in subjects with severe VD deficiency than those with mild and moderate VD deficiency (Figure 1D,E), suggesting that the high bone density observed in the severe VD deficiency group could be attributed to a lower bone turnover.

Measurements of cardiovascular and metabolic risk are displayed in Table 2. Those with severe VD deficiency had significantly higher blood pressure; fasting plasma insulin, the model for insulin resistance; HOMA-IR; triglycerides; cholesterol; and LDL than the two other groups. Due to their role as methyl donors/cofactors in the one-carbon metabolism (OCM) cycle, blood levels of folate and vitamin B12 have been linked to DNA methylation [28,29]. Homocysteine (Hcy), a byproduct of the OCM cycle, was found to be an independent cardiometabolic risk factor that inversely correlated with vitamin B12 and folate [28,30,31]. In this study, we found that the severe VD deficiency group had significantly lower levels of folate and B12 and higher levels of blood Hcy than the other groups. Males and females in the current cohort exhibited significant differences in cardiometabolic risk factors such as HbA1c (mean: M = 6.9, F = 5.6, *p* = 0.0111), HDL (mean: M = 37.6, F = 43.5 mg/dL, *p* = 0.0078), and Hcy (mean: M = 14.3, F = 18.7 µmol/L, *p* = 0.0013). Similarly, African American (AA) participants exhibited statistically significant differences from white (W) participants in terms of total cholesterol (mean: W = 151.9, AA = 168.6, *p* = 0.0308), LDL (mean: W = 84.2, AA = 105.1, *p* = 0.0193), and folate (mean: W = 18.1, AA = 13.9, *p* = 0.0144). Thus, sex and race were accounted for in the comparison between the VD deficiency groups.

### 2.2. Vascular Measurements

**Figure 2:**
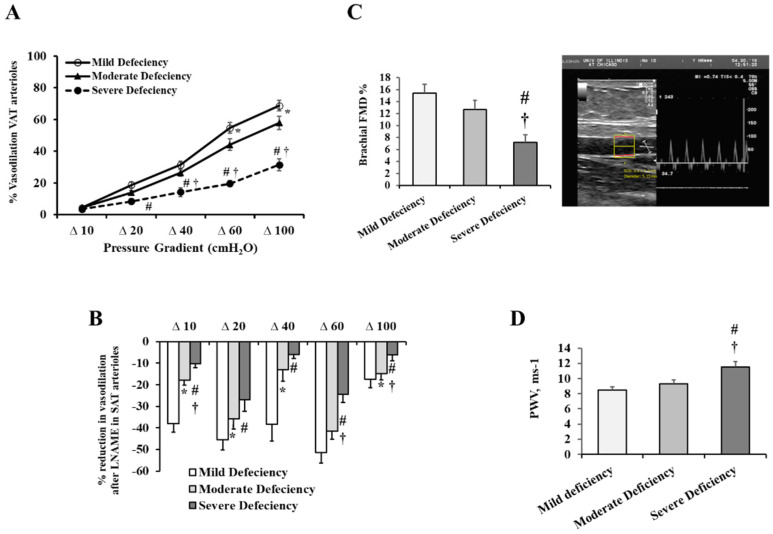
Vascular measurements in different VD deficiency categories. (**A**) FID measurements in VAT-isolated arterioles exposed to increasing intraluminal pressure gradients (10,100 cmH_2_O). (**B**) The percentage decrease in arteriolar FID that was caused by the inhibition of eNOS by L-NAME. (**C**) Quantification chart and representative image for brachial artery FMD measurements using Doppler ultrasound. (**D**) The carotid–femoral pulse wave velocity (PWV) in three VD deficiency groups. Results are shown as means ± standard error (SE). A significant *p*-value is represented by * for comparing the mild and moderate deficiency groups, # for comparing the mild and severe deficiency groups, and † for comparing moderate and severe deficiency groups.

The measured arteriolar flow-induced dilation (FID) was markedly lower in participants with severe VD deficiency than in the two other groups across all pressure gradients (Figure 2A). For the moderate VD deficiency group, significant reductions were only apparent at higher pressure gradients (Δ60 and Δ100 cmH_2_O). The FID was 21% lower in those with substantial VD deficiency compared to those with mild deficiency at a pressure gradient of Δ60 cmH_2_O, which is analogous to the pressure in the arteriolar system of the human body (*p* < 0.0001). LNAME, an inhibitor of endothelial nitric oxide synthase (eNOS), was used to examine the reliance of FID on NO production. Arterioles from the mild deficiency category showed marked reductions in the measured FID in response to LNAME. These reductions were significantly lower in the other groups, especially those with severe VD deficiency (Figure 2B). At Δ60 cmH_2_O, the average FID reductions in response to L-NAME were 78%, 64%, and 30% in the mild, moderate, and severe VD deficiency levels, respectively (*p* = 0.0001). This insensitivity to NO inhibition may reflect an endothelial cell dysfunction.

Brachial artery vasoreactivity was assessed using the Doppler ultrasound (Figure 2C). Hyperemia-induced FMD was 72% lower in subjects with severe VD deficiency compared to the mild deficiency group (*p* < 0.0001). Compared to the mild VD deficiency group, the severely deficient group had a considerably greater pulse wave velocity, a measure of arterial stiffness (12% higher, *p* < 0.001, Figure 2D). Unlike the arteriolar and FID measurements, differences in PWV and brachial artery FMD between the mild and moderate deficiency categories were not statistically significant.

**Figure 3:**
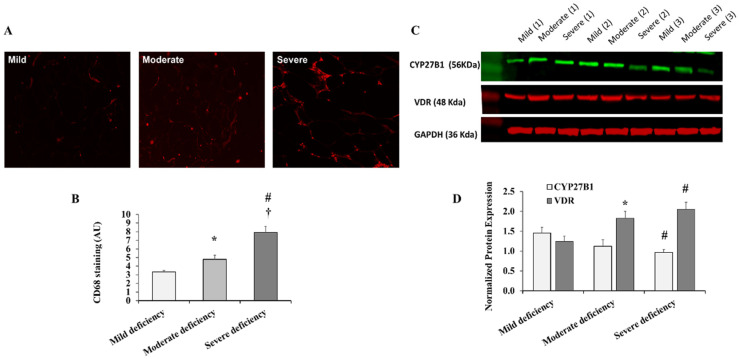
VAT macrophages and protein levels of CYP27B1 and VDR across VD deficiency categories. (**A**) Representative image of CD68 fluorescent staining in VAT from 3 subjects (1 per each VD deficiency group). (**B**) A quantification chart showing the means ± standard error (SE) of the fluorescent signal intensity expressed in arbitrary units (AUs). (**C**) Representative Western blots for analyzing VAT-extracted proteins from 9 subjects (3 per each VD deficiency group). (**D**) A quantification chart showing the means ± standard error (SE) of the target protein signal intensity normalized to the housekeeping gene, GAPDH. A significant *p*-value is represented by * for comparing the mild and moderate deficiency groups, # for comparing the mild and severe deficiency groups, and † for comparing moderate and severe deficiency groups.

The accumulation of macrophages in the adipose tissues is an important factor in obesity-associated inflammation and endothelial dysfunction. Therefore, we sought to test macrophage infiltration in VAT in obese participants from different VD deficiency categories. CD68 positivity, an indicator of macrophage infiltration, was significantly elevated in the VAT of subjects with severe VD deficiency compared to the other two groups (50 to 82%, *p* < 0.001), and moderately elevated in the VAT of those with moderate VD deficiency compared to those with mild VD deficiency (36%, *p* = 0.041) (Figure 3A,B).

Vitamin D has an anti-inflammatory action and an association between VD deficiency and adipose tissue inflammation has been previously reported [32]. The enzyme CYP27B1 (1-alpha hydroxylase) produces the active form of VD, 1,25-dihydroxyvitamin D3, which exerts its effect by binding to the VD receptor (VDR). Therefore, in the present study, we determined the protein concentrations of CYP27B1 and VDR in VAT using Western blotting (Figure 3C,D). The severe VD deficiency group had 40% lower CYP27B1 protein levels than the moderate VD deficiency group (*p* = 0.311), whereas there were no significant differences between the mild and moderate VD deficiency groups. On the other hand, the VDR protein levels increased from mild to moderate (38%) to severe (49%) VD deficiency categories in a dose-dependent manner (*p* = 0.006).

### 2.3. Methylation Modifying Proteins in VAT

**Figure 4:**
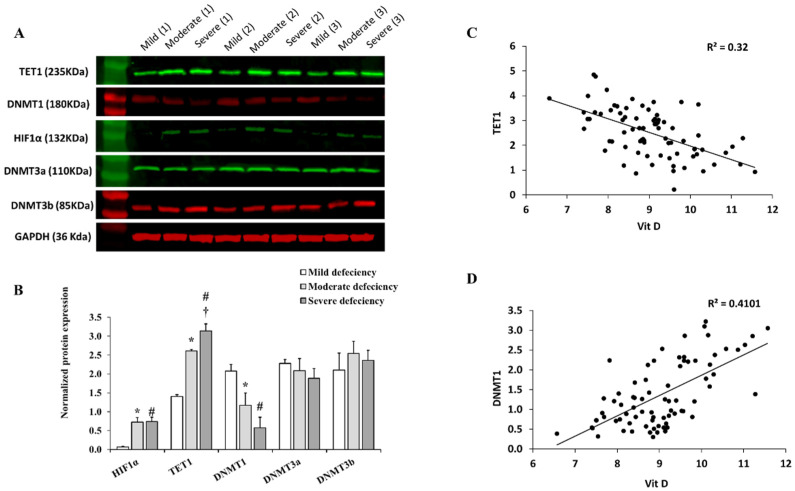
Protein levels of HIF1α, TET1, and DNMTs in VAT across VD deficiency categories. (**A**) Representative Western blots for analyzing VAT-extracted proteins from 9 subjects (3 per each VD deficiency group); all samples were tested but only 9 were presented in this figure. (**B**) A quantification chart showing the means ± standard error (SE) of the target protein signal intensity normalized to the housekeeping gene, GAPDH. A significant *p*-value is represented by * for comparing the mild and moderate deficiency groups, # for comparing the mild and severe deficiency groups, and † for comparing moderate and severe deficiency groups. (**C**,**D**) Scatter plots and *R*^2^ for the correlation between VD concentrations and protein levels of TET1 (**C**) and DNMT1 (**D**).

We previously found that VAT from obese people had reduced vascularization and increased hypoxia, as evidenced by more HIF1α proteins compared to lean controls. We also found that these criteria are mechanistically linked to DNA hypomethylation and increased TET1 protein levels. The current study compared HIF1α and TET1 protein levels in different VD deficiency categories. Other enzymes involved in DNA methylation modification, such as DNA methyltransferases, DNMT1, DNMT3a, and DNMT3b, were also investigated. The HIF1α protein was 1.6–1.7 folds higher in the moderate and severe VD deficiency groups, and the TET1 protein was 60% and 76% higher, respectively, than in the mild deficiency group (Figure 4A,B; *p* < 0.0001). Among the three DNA methyltransferases (DNMTs) tested, only DNMT1 showed statistically significant dose-dependent reductions as the degree of VD deficiency increased (Figure 4A,B). The vitamin D concentrations in obese subjects were negatively correlated with the VAT levels of TET1 (*r* = −0.57; Figure 4C) and positively with the DNMT1 (*r* = 0.64; Figure 4D). These correlations remained significant after controlling for BMI. Altogether, these observations suggest a link between VD deficiency and the process of DNA methylation in the VAT of obese individuals. However, further mechanistic studies are required to confirm this relationship.

### 2.4. DNA Methylation and Inflammatory Biomarkers

**Figure 5:**
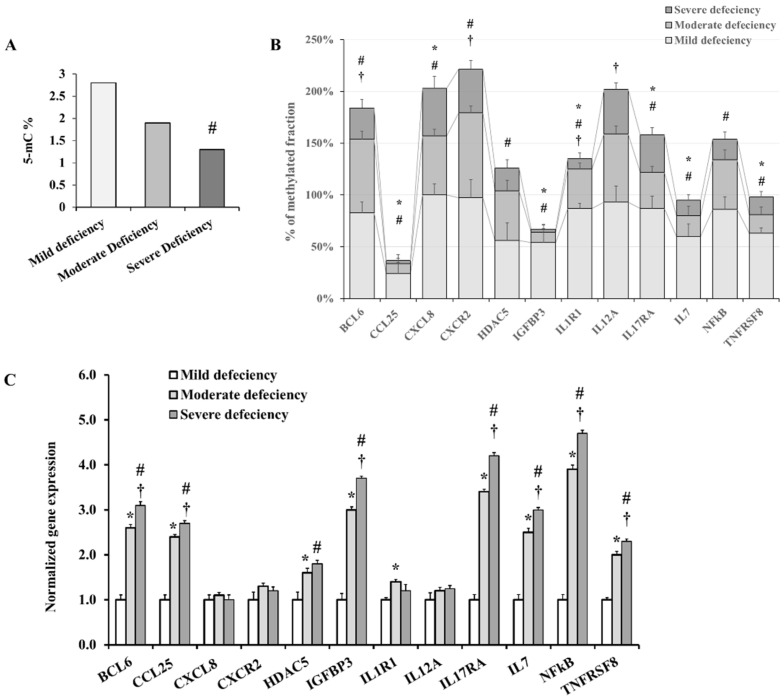
Adipokine promoter methylation and mRNA expression. (**A**) A bar graph showing the relative abundance of 5´-mC in DNA samples extracted from VAT samples. (**B**) A bar chart for the percentage of promoter methylation for each of the 12 differentially methylated adipokines in VAT samples. (**C**) Analysis of the relative abundance of 10 differentially expressed adipokines in VAT tissue samples using qPCR. Results are shown as means ± standard error (SE). A Significant *p*-Value is represented by * for comparing the mild and moderate deficiency groups, # for comparing the mild and severe deficiency groups, and † for comparing the moderate and severe deficiency groups.

The percentage of 5′-methylcytosine (5′-mC) was used to calculate the global levels of DNA methylation in the adipose tissue samples. Subjects with moderate and severe VD deficiencies had 38% and 73% lower 5′-mC than those in the mild deficiency category (Figure 5A). Promoter methylation of inflammation-related genes was measured in the VAT using a commercially available array of 94 inflammatory adipokines. When all genes were averaged together, individuals with moderate and severe VD deficiency had significantly lower levels of methylation than those with a mild deficiency (52%, 30%, and 21%, respectively; *p* < 0.001). It was found that severe VD deficiency had significantly lower promoter methylation levels than moderate deficiency for approximately 70% of the array’s genes. Twelve of the array’s 94 genes exhibited a noticeable variation in promoter methylation among the three VD deficiency categories following correction for multiple comparisons (Figure 5B). Quantitative polymerase chain reaction (qPCR) was used to assess mRNA expression of differentially methylated genes to determine whether the promoter methylation status influences the transcription of these genes. Based on our findings, the mRNA levels of eight out of the twelve proinflammatory genes were significantly higher in the more severe forms of VD deficiency (Figure 5C). The top of these genes are *BCL6*, *CCL25*, *IGFBP3*, *IL17RA*, *IL7*, *NFκB*, and *TNFRSF8* (*p* < 0.0001).

Table 3 presents a summary of the differential expression of these biomarkers, which can be classified, based on their biological function, into four groups, as follows: (1) inflammatory interleukins and TNFα; (2) adhesion molecules (sICAM-1 and sVCAM-1); (3) acute-phase proteins (active PAI-1 and CRP); (4) inflammatory chemokines (sCD40L, CXCL10, and MCP-1).

Individuals with moderate VD deficiency had higher inflammatory biomarkers and adhesion molecules, such as CXCL10, IL6, IL17, active PAI, and sVCAM, than those with mild VD deficiency. Most of the measured inflammatory cytokines, adhesion molecules, and acute-phase proteins were found to be the highest in the subjects with a severe VD deficiency. Nitric oxide (NO) levels in the blood were also determined by measuring nitrates and nitrites and were found to be significantly lower in the higher VD deficiency categories (Table 3).

### 2.5. Vitamin D Deficiency and Cardiometabolic Risk Correlations and Regression Models

**Figure 6:**
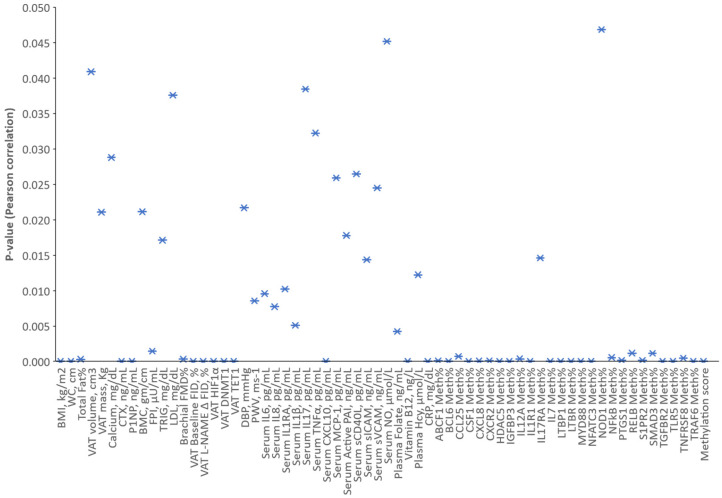
Unadjusted associations between VD levels and cardiometabolic risk factors. A scatterplot for the unadjusted *p*-values of the association between measured VD concentrations in blood and cardiometabolic risk factors using Pearson or Spearman correlations based on the normality of distribution.

Among the variables that were measured in the present cohort of obese individuals, VD concentrations demonstrated statistically significant inverse correlations with BMI, waist circumference, body fat percentage, VAT levels of HIF1α and TET1 proteins, serum CRP and CXCL10 levels, and plasma Hcy concentrations (Figure 6). Significant positive correlations were found between VD levels and brachial artery FMD, arteriolar FID, bone formation/resorption markers (CRX and P1NP), vitamin B12, the methylation levels of several adipokines, and the calculated methylation score (Figure 6). There were modest negative correlations with VAT mass, fasting plasma insulin, triglycerides, LDL, diastolic blood pressure, arterial stiffness (PWV), and serum levels of inflammatory cytokines, chemokines, and adhesion molecules. In addition, there were modest positive correlations with calcium, BMC, plasma folate, and serum NO concentrations. These correlations are summarized in Figure 6, which represents the *p*-values for the unadjusted crude associations between VD blood concentrations and measured cardiometabolic risk factors.

**Figure 7:**
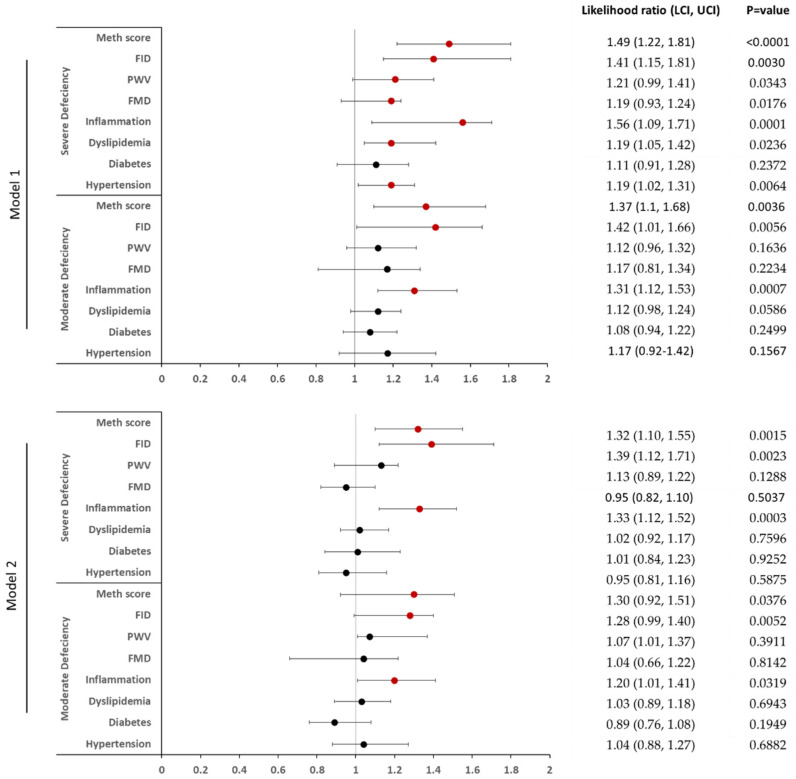
Vitamin D deficiency and the risk of cardiometabolic disease. Regression model 1 accounted for gender, age, and race, and regression model 2 accounted for gender, age, race, and BMI. Hypertension (clinical history or SBP/DBP ≥ 130/80 mmHg); dyslipidemia (triglycerides ≥ 150 mg/dL, total cholesterol ≥ 200 mg/dL, LDL ≥ 100 mg/dL; or HDL < 40 mg/dL); diabetes (clinical history of diabetes or fasting blood glucose ≥ 126 mg/dL); systemic inflammation (CRP > 3.5 mg/dL or IL-6 > 17.5 pg/mL); arteriolar FID (<33.8%); PWV (>10.1 ms^−1^); brachial artery FMD (<8.0%); inflammatory gene methylation score (<43.2%). FID, flow-induced dilation; FMD, flow-mediated dilation; LCI, lower confidence interval; PWV, pulse wave velocity; UCI, upper confidence interval. Red circles indicate significant *p*-values.

Following that, we built logistic regression models that accounted for confounding variables such as age, race, and gender in model 1, and those covariates plus the BMI in model 2. The results of these models show a strong association between VD deficiency and a high cardiometabolic risk (Figure 7). Classifications of diabetes, hypertension, and dyslipidemia were based on the patient’s clinical history or established criteria published by the American Diabetes Association (ADA) and the American Heart Association (AHA). The median values were used to categorize the other continuous variables into two groups, with the mild VD deficiency group serving as the reference group. In order to summarize DNA methylation information, the mean methylation of differentially methylated proinflammatory genes was calculated, yielding a methylation score.

In model 1, the risk of hypertension and dyslipidemia increased by 19% (*p* = 0.006 and 0.02, respectively). The risk of inflammation increased by 31% (*p* = 0.0007) and 56% (*p* = 0.0001) in the moderate and severe VD deficiency categories, respectively. The risk of arterial stiffness and impaired arterial FMD increased in the severe deficiency group by 19% and 21% (*p* = 0.02 and 0.03, respectively). The likelihood of having lower arteriolar FID was ~40% higher for both the moderate and severe deficiency groups (*p* = 0.005 and 0.003, respectively). Finally, compared to the reference group, the risk of having a low methylation score, which translates into higher inflammatory adipokine expression, was 37% and 49% higher in the moderate and severe VD deficiency groups (*p* = 0.003, <0.0001, respectively).

In model 2, which was additionally adjusted for BMI, the association between VD deficiency and variables such as hypertension, dyslipidemia, arterial FMD, and arterial stiffness was lost. However, other variables that continued to show significant associations included inflammation (20% higher in moderate deficiency (*p* = 0.03) and 33% higher in severe deficiency (*p* = 0.0003)), arteriolar FID (28% higher in moderate deficiency (*p* = 0.005) and 39% higher in severe deficiency (*p* = 0.002)), and methylation score (30% higher in moderate deficiency (*p* = 0.03) and 32% higher in severe deficiency (*p* = 0.002)).

## 3. Discussion

Vitamin D is an endogenously synthesized hormone; its active form, 1,25-dihydroxy vitamin D (1,25(OH)2D), regulates bone turnover and contributes to several biological functions, including cell differentiation, proliferation, and immune system modulation. A deficiency in VD is associated with disorders of the cardiovascular, endocrine, immune, and nervous systems [33]. In the blood, clinically relevant concentrations of VD are measured as 25(OH)D; for serum 25(OH)D levels to be considered normal or sufficient, they must be ≥30 ng/mL; serum 25(OH)D concentrations between 21 and 29 ng/mL are deemed insufficient, and concentrations less than 20 ng/mL are considered deficient, as defined by the American Geriatrics Society Workgroup [34].

Vitamin D deficiency is prevalent in obese individuals, and both BMI and visceral fat mass are often used as determining factors of serum VD status [35]. This phenomenon has been noted in adults as well as children and adolescents who suffer from obesity [36,37]. Obesity, particularly visceral obesity, is linked to insulin resistance, metabolic diseases, and cardiovascular diseases. Vitamin D levels were found to be inversely related to weight, BMI, and indicators of type 2 diabetes, including large weight circumference and elevated hemoglobin-A1c (HbA1c) [38,39]. Low VD levels were also associated with various cardiovascular risk parameters, including low-density lipoprotein cholesterol (LDL-C), triglycerides (TGs), systolic blood pressure (SBP), and HbA1c [39]. Studies on the effects of VD supplementation in treating obesity or the accompanying dysmetabolic state have yielded inconsistent results, and it is disputed whether VD deficiency is a cause or consequence of obesity. Some of the hypothesized causes of VD deficiency in obese people include low VD intake due to poor dietary practices, lesser sunlight exposure because there are not enough outdoor activities, deficiencies in the enzymes involved in VD metabolism, and volumetric dilution [33].

Obesity and VD deficiency have been shown to be linked to inflammation. Previous experimental studies have shown that diet-induced VD insufficiency elevated macrophage infiltration and inflammation levels in the adipose tissue of rats. Additionally, VD insufficiency has reduced the activity of sirtuin 1 (SIRT1) and adenosine monophosphate-activated protein kinase (AMPK), both being energy sensors and inflammatory regulators [40]. Gao et al. [41] have shown that the active form of VD interferes with the tumor necrosis factor α (TNFα)-induced low-grade inflammation in murine and human adipocytes. They also observed reductions in proinflammatory cytokines and chemokines, such as interleukin-6 (IL-6) and macrophage chemoattractant protein-1 (MCP-1), which inhibited the migration of monocytes through cultured preadipocytes. Similar findings have been reported in clinical trials where obese individuals exhibited lower levels of IL-1β, MCP-1, and Toll-like receptor 4 (TLR-4) after 12 weeks of VD intake (50,000 IU/week) [42].

Our previous studies demonstrated the efficacy of VD in enhancing vasoreactivity and nitric oxide production by adipose tissue arterioles isolated from obese individuals [21], which is consistent with data indicating the role of VD in decreasing adipose tissue inflammation and oxidative stress [43]. In addition, our previous research demonstrated that the inflammation of adipose tissue in morbidly obese individuals is mediated by DNA hypomethylation of inflammatory adipokines [22,23]. Therefore, we aimed to determine whether VD deficiency was associated with hypomethylation of adipokines, inflammation of adipose tissue, impaired vascular function, and increased cardiometabolic risk in morbidly obese people. Lower levels of VD were significantly correlated with higher BMI and fat percentages, impaired glucose and lipid metabolism, induced systemic and adipose tissue inflammation, decreased adipokine methylation, and diminished vascular reactivity and elasticity. However, after adjusting for BMI, the relationship remained statistically significant only for the reduced methylation score, systemic inflammation, and impaired vasoreactivity of isolated adipose tissue arterioles.

Vitamin D association with reduced adipokine methylation may be attributable to changes in the adipose tissue levels of DNTM1 and TET1, both of which were significantly associated with VD levels even after controlling for BMI. The link between VD and epigenetics is reciprocal; VD plays a part in regulating the epigenome, and the epigenome impacts aspects of VD metabolism and function [25]. However, studies investigating the relationship between VD and DNA methylation primarily focused on cancers and yielded contradictory results. For example, higher VD levels were associated with lower methylation of Wnt Family Member 5A (*WNT5A*) and dickkopf 1 (*DKK1*) genes in colorectal cancer patients and higher methylation of Retinoid X Receptor Alpha (*RXRA*) and NAD Synthetase 1 (*NADSYN1*) genes in breast cancer patients. Despite this disparity at the level of gene methylation, global DNA methylation has demonstrated relatively more consistent results. In a combined cross-sectional and randomized clinical trial, Zhu et al. [44] observed a direct correlation between VD deficiency and global DNA hypomethylation in peripheral blood cells, which was more prevalent among African Americans than Caucasians. In addition, the same study reported dose-dependent increases in global DNA methylation in African Americans after a 16-week VD intake intervention. These results align with our research showing dose-dependent reductions in the percentage of 5-mC as the level of VD deficiency increases in the adipose tissues of obese people. However, we were unable to find any appreciable differences between various racial/ethnic groups in our study.

There is a clear gap in research on the effect of VD on proteins involved in DNA methylation regulation, such as DNMTs and TETs. Due to the cross-sectional design of the current study, it is impossible to infer the direction of the association between VD deficiency and DNA hypomethylation. Nonetheless, the fact that VD levels are strongly associated with DNMT1 and TET1 protein levels after controlling for BMI and other confounders is crucial information that should guide future mechanistic studies testing the interaction between VD and DNA methylation in tissues. In addition to its association with global methylation, VD demonstrated a robust positive association with DNA methylation of inflammatory adipokines in the adipose tissues of obese individuals. Those with severe VD deficiency exhibited the lowest levels of DNA methylation in almost 70% of the examined inflammatory adipokines even after adjusting for BMI; those with a moderate or severe VD deficiency were more likely to have a lower adipokine methylation score and a higher expression of these hypomethylated adipokines. In support of these findings, previous studies have reported the role of VD deficiency in developing subclinical inflammation and immune cell infiltration in the adipose tissues of obese individuals [37,45]. However, this is the first study to our knowledge to link VD deficiency levels to adipokine methylation status in the adipose tissues of morbidly obese individuals.

Although some of the cardiometabolic risk factors, such as body fat percentage, glucose metabolism, and lipid profile, varied between males and females as well as between subjects of white and African American descent, no appreciable variations in the methylation or expression of the examined inflammatory genes were found. This limited impact of sex or race in our study could be attributed to the small sample size. Previous research with larger sample sizes has revealed that individuals from various racial and sex backgrounds have different levels of inflammatory gene expression and methylation. For example, a study by Hall et al. [46] demonstrates gender differences in global DNA methylation and promoter methylation of genes involved in insulin signaling and other metabolic pathways. Similarly, differences in global methylation and the methylation of genes involved in inflammation, metabolism, cardiovascular function, and pain pathways were observed between individuals of different racial/ethnic backgrounds [47,48,49]. The associations between adipose tissue inflammation and systemic inflammation that our laboratory and others have documented suggest a significant contribution of body fat to the mild chronic inflammation seen in obesity [23,50]. In the current investigation, we found a strong correlation between VD deficiency and the levels of circulating inflammatory mediators such as TNFα, CRP, and chemokines, as well as adhesion molecules such as sICAM-1 and sVCAM-1. A systemic inflammation score was calculated based on circulating levels of IL-6 or CRP. Higher levels of VD deficiency were associated with a more significant systemic inflammation score in obese individuals after controlling for age, race, gender, and BMI. These findings are in line with previous studies showing that VD supplementation reduced CRP and TNFα in obese diabetic patients and caused a switch in T-cell phenotype from proinflammatory (Th1/Th17) to anti-inflammatory (Th2/Treg) as well as a shift in the secretory profile from proinflammatory (IL-1β, IL-6, IL-8, IL-12, IL-17, TNFα, and IFNγ) to anti-inflammatory (IL4 and IL10) [51,52].

It is anticipated that the induced infiltration of inflammatory macrophages will accompany the increased levels of inflammatory mediators and cytokines in VAT. In our study, we observed dose-dependent increases in VAT infiltration with macrophages as the degree of VD deficiency increased. These findings are consistent with recent experimental and preclinical studies that reported an effect of VD in inhibiting monocyte chemoattractant protein 1 (MCP-1) and macrophage recruitment in adipose tissues [53,54]. Several studies have supported the anti-inflammatory effects of Vitamin D in adipose tissues and other metabolic tissues [32]. Vitamin D is converted into the active form, 1,25-dihydroxyvitamin D3, by the enzyme CYP27B1 (1-hydroxylase), which acts by binding to VDR. Our current research showed a significant decrease in CYP27B1 in the VAT from subjects with severe VD deficiency, which is consistent with earlier research that suggested the downregulation of CYP27B1 as a contributing factor to VD deficiency in obese individuals [55]. An increase in VDR protein levels was observed as VD levels declined, corroborating previous studies that demonstrated similar patterns of VDR expression in the adipose tissues of morbidly obese subjects compared to normal-weight controls [32,56,57,58]. Although some of these studies were unsuccessful in establishing a link between VDR in adipose tissues and serum levels of active VD, in the current study, VDR levels increased in a dose-dependent manner as the severity of VD deficiency increased.

One of this study’s key findings is the strong correlation between VD deficiency and vascular dysfunction. In addition to an increase in pulse wave velocity, a sign of arterial stiffness, VD deficiency was also linked to impaired vasoreactivity in the brachial artery and adipose tissue-isolated arterioles. Adjusting for BMI eliminated these correlations, except for arteriolar FID, which may be explained by the direct exposure of these arterioles to the adipose tissue inflammatory milieu that was found to be significantly linked to VD deficiency. Several studies have demonstrated VD’s role in lowering cardiovascular risk and maintaining arterial health [59,60]. Still, fewer have examined VD’s impact on microvascular function, partly explained by the difficulties inherent in gauging microvascular reactivity. We used our established ex vivo measurements of vasoreactivity in tissue-isolated arterioles to demonstrate that the decline in microvascular function is dose-dependent on the level of VD deficiency. Additionally, we discovered that VD increased the arteriolar sensitivity to nitric oxide, a finding corroborated by earlier in vitro studies that showed a positive impact of VD on endothelial cell function by promoting NO generation and minimizing inflammation and oxidative stress [61,62].

Vitamin D is necessary for the absorption of calcium and bone turnover; therefore, VD deficiency is likely associated with lower calcium and bone mineral density (BMD) levels. Indeed, our results show that moderate and severe VD deficiencies were associated with lower calcium levels than mild VD deficiency. However, BMD and bone mineral content (BMC) measured by DEXA scanning were significantly higher in individuals with higher levels of vitamin D deficiency and positively correlated with BMI. The relationship between body fat and BMD is controversial, and there is mounting evidence linking obesity and increased bone density. Individuals with a higher body mass tend to have a higher BMD, which is believed to be due to the mechanical effect of weight on bones and the greater amount of estrogen produced by excess fat [63,64]. Despite this apparent increase in bone density, our data showed a significant reduction in bone turnover as obesity and VD deficiency increased. Reductions in C-terminal telopeptide of type 1 collagen (CTX), a bone resorption enzyme, and procollagen type 1 N propeptide (P1NP), a bone formation enzyme, were used as markers for reduced bone turnover in the study participants. Previous studies involving diabetic premenopausal women have produced similar results [65]. However, the clinical significance of bone turnover markers is unclear, and additional research is required to determine whether these are valid markers for impaired bone structure or bone fragility.

In conclusion, this investigation links VD deficiency to molecular and epigenetic findings in adipose tissues and systemic inflammation and vascular function in morbidly obese people. This study offers a distinctive data set suggesting VD deficiency as a potential mediator between obesity and cardiometabolic risk. This study can also be credited with a number of valuable contributions in various respects, including the recruitment of a heterogeneous sample of subjects, namely males and females of varying ages and racial backgrounds. Furthermore, we opted to evaluate the methylation of a set of inflammatory adipokines in surgically obtained adipose tissue samples as opposed to readily available blood samples, which had shown inconsistent results in other investigations. The mRNA expression of these genes was also analyzed to assess the functional impact of the reduced adipokine methylation. Vitamin D deficiency was also correlated with a battery of important cardiometabolic risk factors. These variables included promoter methylation and mRNA expression of adipokines, vasoreactivity measurements both in vivo and ex vivo, and various cardiovascular and metabolic biomarkers. Lastly, we assessed the function of adipose tissue arterioles expected to be immediately affected by the molecular environment in the adipose tissue, as opposed to macrovessels such as the femoral and brachial arteries, which were the focus of previous studies. This approach makes the present study significant from a translational standpoint, since peripheral vascular resistance and blood pressure are regulated mainly by resistance arterioles.

However, there are some shortcomings of this study that should be considered. First, the limited sample size made it more difficult to detect statistically significant changes in most of the examined genes, especially when multiple testing was taken into account. It is possible that the small sample size played a role in dampening the association between VD deficiency and the wide variety of cardiometabolic risk factors that were investigated. Additionally, although the goal of targeting inflammation-related mechanisms was successfully attained, testing only a small subset of adipokines hinders our ability to explore other potentially important pathways between VD deficiency levels. In light of this, more comprehensive DNA methylation arrays will be required for future studies of adipokine methylation in obesity. Lastly, this study is cross-sectional, and the direction of the association between VD deficiency and cardiometabolic risk in obese people cannot be inferred from these data. Accordingly, there is a pressing need for additional research combining longitudinal and interventional methods (clinical trials) to gain a deeper comprehension of the dynamics underlying this relationship. In addition, in order to differentiate the impact of VD deficiency from that of obesity, it will be necessary for future research to examine the association between VD deficiency and cardiometabolic risk in non-obese individuals.

## 4. Materials and Methods

### 4.1. Study Participants

Participants (*n* = 77) who were obese and were scheduled to undergo weight loss surgery at the UI Hospital were recruited to participate in the study. This study only included premenopausal women. Smokers, pregnant women, people with preexisting cardiac, hepatic, or renal conditions, and anyone older than 50 were disqualified. Patients were given information about the study and asked to sign a consent form if they were interested in participating during their clinical visit. All recruitment, screening, and consenting documents used in this study conformed to the most recent version of the Declaration of Helsinki and were approved by our Institutional Review Board. One week before surgery, participants underwent a data and sample collection visit at the University’s Clinical and Translational Center, where anthropometric measures and blood specimens were collected, and a brachial artery ultrasound was performed. Study participants had samples of visceral adipose tissue (VAT) obtained from them. Microvessel isolation and dissection were performed using a part of the collected fat samples. The remaining adipose tissue samples were stored in liquid nitrogen for biological analysis.

### 4.2. Body Composition, Cardiovascular, and Metabolic Measures

Body mass index (BMI) and subject weight were among the physical characteristics evaluated. The mass of total and visceral adipose tissue, lean muscle mass, and bone mineral density were all measured with a DXA (dual X-ray absorptiometry) scan (iDXA, General Electric Inc., Boston, MA, USA). We used our standard methods to measure fasting plasma insulin and glucose [28]. Fasting insulin (U/L) x fasting glucose (nmol/L)/22.5 was used to determine the insulin resistance homeostasis model assessment (HOMA-IR) [66]. Measurements of lipids, including cholesterol, high- and low-density lipoproteins, and triglycerides, were performed using Roche Diagnostics enzyme assays (Indianapolis, IN, USA), as described in our previous publications [22,23,28]. In order to measure the hemoglobin A1c (HbA1c), we used a kit from BioVision Inc. (Waltham, MA, USA) [22,23,28]. Nitrate and nitrite, two NO metabolites, were measured using a Nitrate/Nitrite Colorimetric Assay Kit to infer the NO concentration in the serum samples (Cayman Chemicals, Ann Arbor, MI, USA) [21,67,68]. In this assay, nitrates were converted into nitrites using the provided nitrate reductase enzyme, followed by transforming all the nitrites in the samples into dark purple azo molecules using the provided Griess reagents. The color intensity was measured at 540 nm using a plate reader (Molecular Devices, San Jose, CA, USA).

The C-reactive protein (CRP) in blood samples was quantified using CRP ELISA Kit from R&D Systems (Minneapolis, MN, USA); samples, controls, and standards were incubated in microplates precoated with a specific CRP antibody, followed by incubation with an enzyme-linked secondary antibody that binds CRP. The substrate solution was added, and color development was stopped using the supplied stop solution. Lastly, CRP concentrations were measured using a multimode plate reader to determine the optical density at 450 nm. Human C-terminal cross-linking telopeptide of type I (CTX-1) and human procollagen type 1 N-terminal propeptide (P1NP) ELISA Kits (Novus Biologicals, Centennial, CO, USA) were used to measure plasma levels of CTX1 and P1NP. In these assays, samples, controls, and standards were added to a primary antibody-coated plate (90 min at 37 °C) followed by biotinylated detection antibody working solution (60 min), HRP conjugate working solution (30 min), substrate reagent (15 min), and finally, stop solution. The color intensity was assessed using a microplate reader set to 450 nm.

Circulating inflammatory assays, including interleukins (IL1β, IL1RA, IL6, IL8, IL10, and IL17), tumor necrosis factor-alpha (TNFα), plasminogen activator inhibitor (PAI), soluble CD40 ligand (CD40L), monocyte chemoattractant protein-1 (MCP-1), intracellular adhesion molecule (ICAM), and vascular cell adhesion molecule (VCAM) levels in plasma were determined using High Sensitivity Magnetic Luminex Performance Assays (R&D). The samples were first incubated with a biotinylated antibody and a streptavidin-phycoerythrin conjugate and then with magnetic microparticles conjugated with the primary antibody. A Luminex MAGPIX Instrument System was used for analyzing the analytes after the microparticles were reconstituted (Thermo Fisher Scientific, Waltham, MA, USA). The UI Hospital laboratory performed the standard analysis of a complete blood count and liver and kidney function parameters.

### 4.3. Measuring Folate, Vitamin D, Vitamin B12, and Homocysteine (Hcy)

Using a Hcy-specific ELISA assay (Cell Biolabs Inc., San Diego, CA, USA), we were able to determine the concentration of Hcy in the plasma [28]. Samples were first incubated on microplates coated with Hcy at RT for 15 min before being incubated with the primary anti-Hcy antibody, followed by the specific secondary antibody. After waiting 30 min, we added the substrate solution, and then color development was stopped using the stop solution. The absorbance of each sample was measured at 450 nm using a BioRad iMark Absorbance Microplate Reader (Hercules, CA, USA). The Roche Diagnostics Folate III kit (Indianapolis, IN, USA) was used to determine the concentrations of folate and vitamin B12. Vitamin D was measured using 25 (OH) Vitamin D competitive ELISA kits from Creative Diagnostics (Shirley, NY, USA) following the recommended protocol that included (1) incubation of samples, controls, and standards in an antibody-coated plate; (2) addition of labeled antigen conjugated with biotin; (3) incubation with HRP labeled streptavidin; (4) washout of the unbound antigen; (5) utilization of substrate and stop solution; and finally measuring the absorbance at 450 nm using a plate reader.

### 4.4. In Vivo Vasoreactivity Measurements

Using the Hitachi Prosound Alpha 7 (Hitachi Aloka Medical America, Wallingford, CT, USA), we assessed vasoreactivity in the brachial artery [28]. A cuff was placed around the middle of the arm and inflated to 220 mmHg for five minutes. The ultrasonography probe was positioned proximally to the antecubital fossa. The arterial diameter was recorded 60 s before the cuff was inflated (baseline) and 300 s after deflation (reactive hyperemia). Images were taken by the Automated Edge Detection software. In order to identify the relative flow-mediated dilation (FMD), the largest diameter achieved at baseline was subtracted from the largest diameter achieved following the deflation of the cuff (FMD = (brachial artery diameter during hyperemia in mm–brachial artery diameter at baseline in mm)/(baseline diameter in mm × 100)) [21,22,28,68,69]. Using the SphygmoCor program (AtCor Medical, Sydney, Australia) and an applanation tonometer (Millar Instruments, Houston, TX, USA), we calculated the pulse wave velocity by comparing the waveforms at the femoral and carotid arteries.

### 4.5. Vasoreactivity of AT-Isolated Arterioles

Fat tissues obtained from obese individuals were dissected to isolate the embedded small resistance microvessels, which were then cleaned and mounted in an organ perfusion chamber. The internal diameters were measured under increasing pressure gradient following our previously published protocol [21,22,67,68,70]. The diameter of the cannulated arterioles was then visualized using video microscopy to observe arteriolar vasoreactivity under controlled conditions of temperature (37 °C), pH (7.4), and aeration (a mixture of O_2_ (21%), CO_2_ (5%), and N_2_ (74%)). After initial measurements were taken, the ability of arterioles to constrict in response to angiotensin II (10^−6^ mol/L) was tested, and only vessels that were constricting by more than 30% were used for further measurements. Arteriolar sensitivity to nitric oxide was measured by adding the endothelial NO synthase inhibitor L-NAME (10^−4^ mol/L). Arteriolar FID was measured as a percentage by comparing the diameter of the arteries before and after angiotensin II administration in each pressure gradient.

### 4.6. Global DNA Methylation Analysis

In order to estimate the global levels of DNA methylation, the percentage of 5′-methyl cytosine (5′-mC) in the VAT-extracted DNA was measured using a 5′-mC quantifying Kit (Epigentek, Farmingdale, NY, USA). The manufacturer protocol was followed, which includes steps of DNA denaturation, incubation with the provided capture (anti-5′-mC antibody), and detection antibodies (5′-mC Detection Complex Solution), as well as, finally, incubation with the color developer and measuring the color intensity at a wavelength of 405–450 nm using a plate reader as we previously described [23]. The 5′-mC percentage was calculated via the provided standard curve and was proportional to the optical density.

### 4.7. Methylation-Specific PCR (Polymerase Chain Reaction)

This study used EpiTect Methyl II PCR Arrays to examine promoter methylation in a panel of 94 inflammatory and immune-related genes (Qiagen, Chatsworth, CA, USA). Following published methods, mature adipocytes were isolated from visceral adipose tissue (VAT) by enzymatic digestion with 0.1% collagenase type I [71,72]. Adipocyte DNA was extracted using a DNA extraction kit from Qiagen, and its quantity and integrity were determined using a spectrophotometer (Thermo Fisher Scientific, Waltham, MA, USA). The extracted DNA was further processed using the restriction enzymes in the kit. This reaction was carried out by heating mixtures of 2 g of extracted DNA and either methylation-sensitive (Ms) or methylation-dependent (Md) enzymes for 6 h at 37 °C, followed by heat inactivation for 20 min at 65 °C. After that, PCR arrays were conducted in accordance with the manufacturer’s protocol. Qiagen’s MethylScreenTM technology, which is implemented as an Excel sheet, allows for the automated determination of the array gene promoter methylation by means of Ct values.

### 4.8. Quantitative PCR

RNA isolation from fat samples was performed using an RNA extraction kit from Qiagen (Germantown, MD, USA). The extracted RNA was analyzed for quality and quantity using NanoDrop Spectrophotometer, then cDNA molecules were synthesized by using iScriptTM Supermix for RNA reverse transcription, and gene expression was measured quantitatively via qPCR assays. Primers for PCR were developed by Invitrogen Life Technologies and designed using the software Primer3. Gene expression levels were adjusted using the housekeeping gene GAPDH and the Livak method (2^−∆∆Ct^) [73].

### 4.9. Western Blotting

RIPA lysis buffer containing the common protease and phosphatase enzymes (Sigma-Aldrich, St. Louis, MO, USA) was used to extract total protein from samples. For protein quantification, Thermo Fisher Scientific Pierce BCA Protein Assay (Waltham, MA, USA) was utilized, and calculations were made to use 10 μg of the total VAT protein for gel electrophoresis. We followed the steps we published in previous publications [21,22,23]. Primary antibodies included HIF1α rabbit mAb, DNMT1 mouse mAb, DNMT3a rabbit mAb, DNMT3b mouse mAb, TET1 rabbit mAb, VDR mouse mAb, and CYP27B1 rabbit mAb, all purchased from Abcam (Waltham, MA, USA). The housekeeping protein, GAPDH mouse mAB (cell signaling), was used as a loading control. Target proteins were then quantified via Image Studio (LI-COR) and normalized to the GAPDH.

### 4.10. Immunofluorescent Staining of VAT

Paraffin blocks of VAT were sectioned (5 μm) and processed for staining as we previously described [23]. This was followed by immunofluorescent staining with the macrophage biomarker CD68. Briefly, sections were incubated with blocking buffer for 60 min, followed by a primary mouse monoclonal CD68 antibody (1:100, cell signaling) overnight at 4 °C followed by washing steps and incubation with a secondary Alexa Fluor-conjugated antibody (1:1000) for 60 min. Sections were imaged using ZEISS LSM 710 confocal microscope (Carl Zeiss Meditec, Inc. Dublin, CA, USA). Immunofluorescence was quantified using ImageJ software and presented in arbitrary units (AUs).

### 4.11. Statistical Analyses

The distribution of data for all the included variables was tested. We used ANOVA, Kruskal–Wallis, or chi-square tests to analyze normally distributed continuous variables, non-normally distributed continuous variables, and categorical variables, respectively, across different VD deficiency categories. We presented our results as mean ± standard error (SE), and the data were considered statistically significant when the *p*-value was less than 0.05. The *p*-value for multiple comparisons was adjusted using the Bonferroni method (*p* < 0.0003). In order to compute the methylation score, we used the average methylation percentage of differentially methylated genes. Correlations between continuous and categorical variables were tested using bivariate Pearson/Spearman correlation and χ^2^ tests, respectively. VD deficiency was independently linked to cardiometabolic risk factors using multiple regression analysis. Age, race, and gender were taken into consideration in the first model, while body mass index was taken into account in the second model. We used the median value to create a binary classification for the continuous variables, with mild VD deficiency serving as the reference category. SPSS version 28 (SPSS Inc., Chicago, IL, USA) was used to conduct the analyses.

## Figures and Tables

**Table 1:** Anthropometric and bone mass measurements.

Variable	Mild Deficiency (*n* = 21)	Moderate Deficiency (*n* = 30)	Severe Deficiency (*n* = 26)	*p*-Value
Age, y	38.9 ± 2.3	36.4 ± 1.3	34.3 ± 0.2	0.1473
Gender (♀)	15	17	14	0.4303 ^¥^
Race/ethnicity (AA)	10	16	19	0.1629 ^¥^
**Vitamin D and Ca^2+^ measurements**				
25-hydroxyvitamin D, ng/mL	13.5 ± 0.4	9.9 ± 0.1 *	4.7 ± 0.2 *†	<0.0001
Calcium, mg/dL	9.8 ± 0.1	9.4 ± 0.1 *	9.2 ± 0.1 *	0.0006
Phosphorous, mg/dL	3.4 ± 0.1	3.3 ± 0.2	3.1 ± 0.1	0.4050
**Anthropometric DEXA measurements**				
Weight, kg	94.9 ± 5.2	101.2 ± 11.5	148.6 ± 6.0 *†	<0.0001
WC, cm	82.1 ± 7.7	106.7 ± 6.2 *	134.9 ± 4.1 *†	<0.0001
BMI, kg/m^2^	34.1 ± 4.2	34.5 ± 1.9	52.9 ± 1.7 *†	<0.0001
BSA	2.1 ± 0.1	2.2 ± 0.1	2.6 ± 0.1 *†	0.0022
Fat %	34.3 ± 1.5	45.0 ± 2.6 *	53.1 ± 1.6 *†	<0.0001
Lean %	63.1 ± 3.7	53.4 ± 2.4 *	45.9 ± 1.5 *	0.0001
Android fat %	34.3 ± 1.9	47.1 ± 2.7 *	56.2 ± 1.5 *†	<0.0001
VAT mass, kg	0.8 ± 0.3	1.8 ± 0.2 *	2.7 ± 0.3 *†	0.0003
**Bone density measurements**				
BMC, cm^2^	2801.3 ± 91.0	3172.9 ± 100.5	3578.0 ± 163.7 *†	0.0004
BMD, gm/cm^2^	1.3 ± 0.1	1.3 ± 0.03	1.5 ± 0.03 *†	0.0134
T-score	1.8 ± 0.4	3.0 ± 0.2 *	3.6 ± 0.3 *	0.0004
Z-score	0.6 ± 0.1	1.5 ± 0.3	1.6 ± 0.3 *	0.0314

^¥^ for comparing the gender and race/ethnicity among VD deficiency categories using the chi-square test; AA refers to individuals of African American descent; * for comparing variables to the mild VD deficiency category using one-way analysis of variance; † for comparing the severe versus moderate VD deficiency categories with one-way analysis of variance; BMD, bone mineral density; BMC, bone mineral content; BMI, body mass index; BSA, body surface area; CTX, carboxy-terminal cross-linked telopeptide of type 1 collagen; P1NP, propeptide of type 1 procollagen; PLT, VAT, visceral adipose tissues; WC, waist circumference.

**Table 2:** Cardiometabolic health measurements.

Variable	Mild Deficiency (*n* = 21)	Moderate Deficiency (*n* = 30)	Severe Deficiency (*n* = 26)	*p*-Value
**Cardiometabolic measurements**				
FPI, µU/mL	10.1 ± 1.2	11.9 ± 0.9	15.2 ± 0.8 *†	0.0018
FPG, mg/dL	93.5 ± 3.7	97.7 ± 3.9	101.2 ± 3.2	0.3749
HOMA-IR	2.4 ± 0.4	3.4 ± 0.7	3.9 ± 0.4 *	0.0233
HbA1c, %	5.4 ± 0.1	5.5 ± 0.2	5.9 ± 0.3	0.2737
Chol, mg/dL	159.0 ± 4.9	163.8 ± 5.3	179.1 ± 4.8 *	0.0213
LDL, mg/dL	90.9 ± 5.8	93.0 ± 5.3	113.2 ± 4.8 *†	0.0070
HDL, mg/dL	48.7 ± 1.9	47.2 ± 1.8	43.2 ± 1.2	0.0690
Trig, mg/dL	97.7 ± 2.8	112.2 ± 4.1	116.3 ± 4.5 *†	0.0079
HR, bpm	76 ± 4	78 ± 2	82 ± 2	0.2866
Systolic BP, mmHg	123 ± 2	125 ± 2	132 ± 3 *	0.0292
Diastolic BP, mmHg	77 ± 2	78 ± 2	84 ± 1 *†	0.0132
**Other measurements**				
Folate, ng/mL	16.6 ± 1.4	18.6 ± 0.7	13.5 ± 0.9 *†	0.0010
Vitamin B12, ng/L	564.5 ± 36.4	524.5 ± 26.5	323.8 ± 18.2 *†	<0.0001
Hcy, µmol/L	10.2 ± 1.2	12.3 ± 1.3	23.5 ± 3.1 *†	<0.0001

* For comparing variables to the mild VD deficiency category and † for comparing the severe versus moderate VD deficiency categories via one-way analysis of variance; Chol, cholesterol; BP, blood pressure; FPG, fasting plasma glucose; HbA1c, glycosylated hemoglobin; Hcy, homocysteine; FPI, fasting plasma insulin; HR, heart rate; HOMA-IR, homeostatic model assessment for insulin resistance; HDL, high-density lipoprotein; LDL, low-density lipoprotein; Trig, triglycerides.

**Table 3:** Circulating biomarkers of inflammation and vascular function.

Variable	Mild Deficiency (*n* = 21)	Moderate Deficiency (*n* = 30)	Severe Deficiency (*n* = 26)	*p*-Value
**Circulating biomarkers of inflammation and vascular function**				
CRP, mg/dL	1.8 ± 0.8	1.9 ± 0.3	4.4 ± 0.3 *†	0.0001
CXCL10, pg/mL	82.2 ± 7.7	135.2 ± 13.1 *	211.6 ± 16.8 *†	<0.0001
IL1β, pg/mL	11.5 ± 1.4	15.6 ± 2.1	19.0 ± 1.7	0.0295
IL1RA, pg/mL	61.8 ± 7.4	81.2 ± 6.5	84.1 ± 8.1	0.0962
IL6, pg/mL	9.1 ± 0.3	13.0 ± 0.7 *	20.3 ± 1.5 *†	<0.0001
IL8, pg/mL	40.2 ± 3.8	38.7 ± 2.2	53.6 ± 3.9 *†	0.0025
IL10, pg/mL	39.0 ± 1.2	42.4 ± 3.3	38.3 ± 3.1	0.5471
IL17, pg/mL	69.6 ± 3.2	86.3 ± 3.0 *	126.4 ± 7.0 *†	<0.0001
TNFα, pg/mL	19.7 ± 1.1	23.1 ± 2.0	30.7 ± 2.4 *†	0.0012
MCP-1, pg/mL	307.0 ± 12.3	332.0 ± 16.4	370.5 ± 13.5 *	0.0166
Active PAI, ng/mL	21.7 ± 2.0	27.9 ± 1.3 *	29.3 ± 1.7 *	0.0061
sCD40L, pg/mL	346.8 ± 19.1	401.4 ± 21.0	448.7 ± 25.5	0.0125
sICAM-1, ng/mL	116.8 ± 4.8	124.9 ± 6.7	220.3 ± 5.4 *†	<0.0001
sVCAM-1, ng/mL	776.6 ± 11.7	851.1 ± 12.2 *	920.7 ± 13.5 *†	<0.0001
NO, µmol/L	7.9 ± 0.9	5.9 ± 0.4 *	4.1 ± 0.4 *†	0.0001

* For comparing variables to the mild VD deficiency category. † For comparing the severe versus moderate VD deficiency categories; CXCL10, C-X-C motif chemokine ligand 10; CRP, C-reactive protein; IL1β, interleukin 1 beta; IL1RA, interleukin 1 receptor antagonist; IL6, interleukin 6; IL8, interleukin 8; IL10, interleukin 10; IL17, interleukin 17; MCP-1, monocyte chemoattractant protein-1; NO, nitric oxide; sCD40L, soluble CD40 ligand; PAI, plasminogen activator inhibitor-1; sICAM-1, soluble intercellular adhesion molecule-1; sVCAM-1, soluble vascular cell adhesion molecule-1; TNFα, tumor necrosis factor-alpha.

## Data Availability

The article contains all of the findings obtained from the study.
